# Antiprotozoal Effect of Saponins in the Rumen Can Be Enhanced by Chemical Modifications in Their Structure

**DOI:** 10.3389/fmicb.2017.00399

**Published:** 2017-03-16

**Authors:** Eva Ramos-Morales, Gabriel de la Fuente, Stephane Duval, Christof Wehrli, Marc Bouillon, Martina Lahmann, David Preskett, Radek Braganca, Charles J. Newbold

**Affiliations:** ^1^Institute of Biological, Environmental and Rural Sciences, Aberystwyth UniversityAberystwyth, UK; ^2^DSM Nutritional Products Ltd., Centre de Recherche en Nutrition AnimaleSaint Louis Cedex, France; ^3^School of Chemistry, Bangor UniversityBangor, UK; ^4^BioComposites Centre, Bangor UniversityBangor, UK

**Keywords:** antiprotozoal activity, *Hedera helix*, hederagenin, saponins, stability

## Abstract

The antiprotozoal effect of saponins is transitory, as when saponins are deglycosylated to the sapogenin by rumen microorganisms they become inactive. We postulated that the substitution of the sugar moiety of the saponin with small polar residues would produce sapogen-like analogs which might be resistant to degradation in the rumen as they would not be enzymatically cleaved, allowing the antiprotozoal effect to persist over time. In this study, we used an acute assay based on the ability of protozoa to break down [^14^C] leucine-labeled *Streptococcus bovis* and a longer term assay based on protozoal motility over 24 h to evaluate both the antiprotozoal effect and the stability of this effect with fifteen hederagenin *bis*-esters esterified with two identical groups, and five cholesterol and cholic acid based derivatives carrying one to three succinate residues. The acute antiprotozoal effect of hederagenin derivatives was more pronounced than that of cholesterol and cholic acid derivatives. Modifications in the structure of hederagenin, cholesterol, and cholic acid derivatives resulted in compounds with different biological activities in terms of acute effect and stability, although those which were highly toxic to protozoa were not always the most stable over time. Most of the hederagenin *bis*-esters, and in particular hederagenin *bis*-succinate (TSB24), hederagenin *bis*-betainate dichloride (TSB37) and hederagenin *bis*-adipate (TSB47) had a persistent effect against rumen protozoa *in vitro*, shifting the fermentation pattern toward higher propionate and lower butyrate. These chemically modified triterpenes could potentially be used in ruminant diets as an effective defaunation agent to, ultimately, increase nitrogen utilization, decrease methane emissions, and enhance animal production. Further trials *in vivo* or in long term rumen simulators are now needed to confirm the *in vitro* observations presented.

## Introduction

The manipulation of the rumen microbial ecosystem using plant secondary compounds has proved to be a useful strategy to increase the efficiency of feed utilization by ruminants (Bodas et al., 2012; Wanapat et al., 2012). Plants or their extracts with high concentrations of saponins appear to have the potential to act as natural antiprotozoal agents (Patra and Saxena, 2009a). Protozoa are a normal but non-vital part of the rumen microbiome and can contribute up to 50% of the bio-mass in the rumen (Williams and Coleman, 1992). Because of their predation activity, rumen protozoa have been shown to be highly active in the turnover of bacterial protein in the rumen (Wallace and McPherson, 1987). Moreover, protozoa have been proven to harbor an active population of methanogenic archaea both on their external and internal surfaces (Finlay et al., 1994; Newbold et al., 1995). A recent meta-analysis has shown that the elimination of protozoa from the rumen could increase microbial protein supply to the host by up to 30% and reduce methane production by up to 11% (Newbold et al., 2015).

Saponins are plant secondary metabolites which consist of one or more sugar moieties glycosidically linked to a less polar aglycone or sapogenin (Francis et al., 2002). The sugar portion is generally made up of common monosaccharides, such as D-glucose, D-galactose, D-glucuronic acid, D-xylose, L-rhamnose, and various pentoses which are glycosidically linked as linear or branched oligosaccharides to the sapogenin. Saponins can be broadly classified based on their sapogenin structure as either triterpenoid or steroid saponins (Wina et al., 2005). The presence of different substituents in the sapogenin such as hydroxyl, hydroxymethyl, carboxyl, and acyl groups, as well as differences in the composition, linkage and number of sugar chains accounts for significant structural variation and thus their bioactivity (Patra and Saxena, 2009b; Podolak et al., 2010).

Saponins can form irreversible complexes with cholesterol in the protozoal cell membrane causing cell rupture and lysis (Wina et al., 2005). Rumen protozoal species seems to differ in their sensitivity to saponins due to differences in the sterol composition of their cellular membranes leading to the suggestion that feeding saponins might lead to partial defaunation (Patra and Saxena, 2009a). The antiprotozoal effect of saponins is, however, transitory as when saponins are deglycosylated by rumen microorganisms to the sapogenin they become inactive (Newbold et al., 1997; Patra and Saxena, 2009a) which represents a challenge to their practical application in ruminant nutrition. We hypothesized that the substitution of the sugar moiety of the saponin with small polar residues would produce sapogen-like analogs which might be resistant to degradation in the rumen as they would not be enzymatically cleaved, allowing the antiprotozoal effect to persist over time. The aim of this study was to evaluate both the acute anti-protozoal action and the stability of the antiprotozoal effect of chemically synthesized hederagenin, cholesterol, and cholic acid derivatives *in vitro.*

## Materials and Methods

### Hederagenin, Cholesterol, and Cholic Acid Derivatives

Ripe ivy (*Hedera helix*) fruits were collected from several locations around Bangor (44.8036° N, 68.7703° W, UK), dried at 50°C for 2 days and milled. Ivy fruit meal (3.79 kg) was extracted with ethanol (15 L) for 6 h, leading to a crude extract (541 g) comprising triglycerides, saponins, oligosaccharides, and pigments (anthocyanins). The crude extract was then washed with petroleum ether (3 × 500 mL) and dried overnight at 50°C under vacuum, obtaining a fine powder (368 g) which comprised mainly mixed saponins and oligosaccharides. Then an additional extraction with *n*-butanol was carried out, obtaining a refined extract comprising saponins (15% DM). Hederagenin, the aglycone part of the saponins, was obtained via hydrolysis of ivy fruit refined extract in ethanolic solution with aqueous HCl.

Hederoside B, the major saponin present in the fruit extract, was obtained by gravity chromatography (Fluorochem, silica gel 40–60, CHCl_3_/MeOH/H_2_O; 90:9:1 → 75:22.5:2.5) of the defatted fruit extract. Fractions containing hederoside B were concentrated and subsequently washed with methanol. Nuclear magnetic resonance data (pyridine-d_5_) of the obtained compound was in agreement with that reported in the literature (Kizu et al., 1985).

**FIGURE 1:**
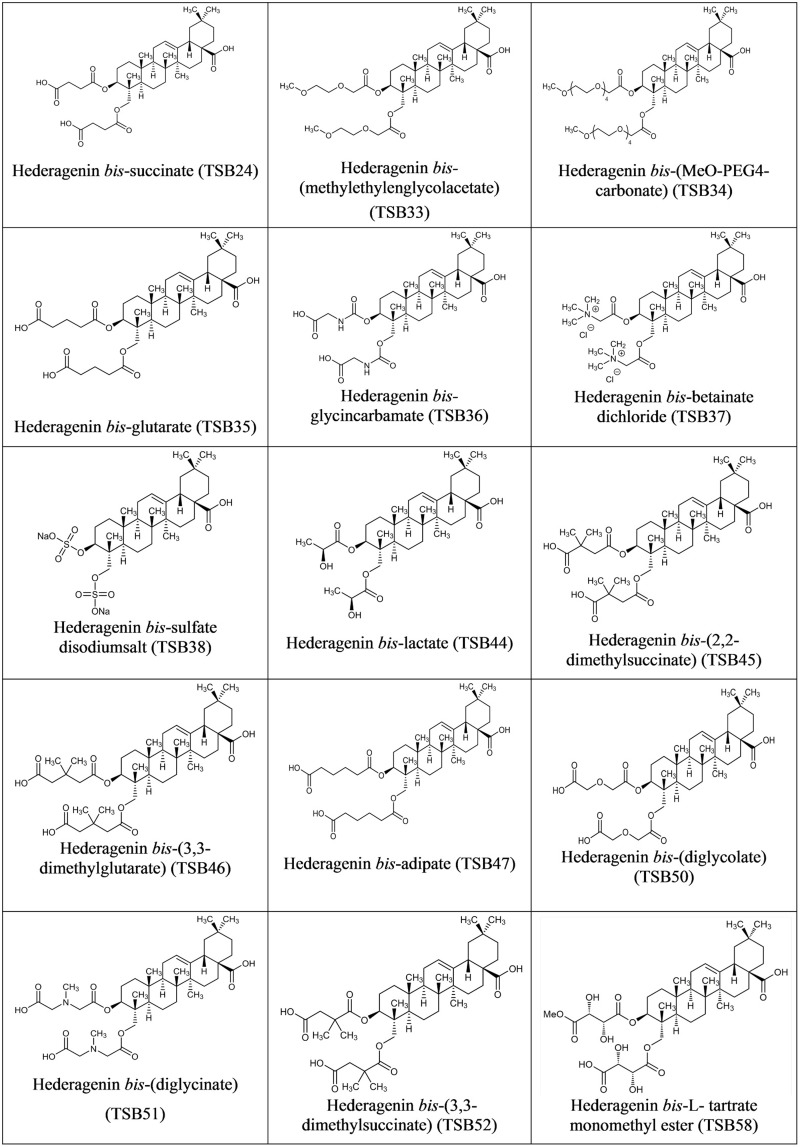
**Structure of Hederagenin derivatives**.

Hederagenin *bis*-esters derivatives (two identical ester moieties at position 3 and 23; **Figure 1**) were synthesized from the aglycone hederagenin produced above as described in patent application PCT/EP2016062383 (Ramos-Morales et al., 2016).

**FIGURE 2:**
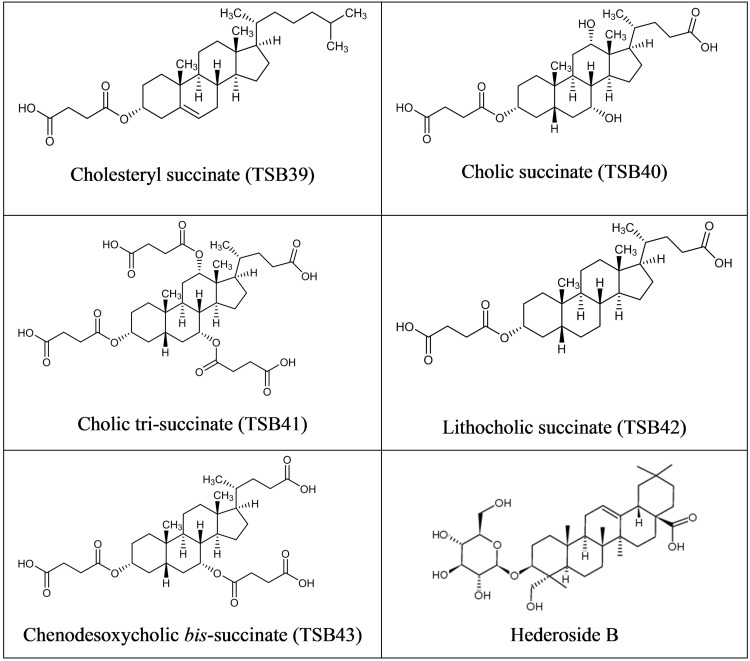
**Structure of Cholesterol and Cholic acid derivatives and Hederoside B**.

Cholesterol and cholic acid derivatives (**Figure 2**) were synthesized following the same methods for esterification of organic molecules, described in patent PCT/EP2016062383 (Ramos-Morales et al., 2016). Hederagenin, cholesterol, and cholic acid derivatives were produced by DSM Nutritional Products and Bangor University.

The purity of the synthesized compounds was established by quantitative nuclear magnetic resonance (qNMR) spectroscopy using a Bruker Ultrashielded 400 spectrometer (Bruker Corporation, Coventry, UK) confirming purities of 80–99% for most derivatives except TSB37 and TSB38 which had a purity of 66 and 58%, respectively. It should be noted that the antiprotozoal activity of compounds TSB37 and TSB38 may be either over or indeed underestimated due to the impurities present.

### Measurement of Protozoal Activity

The effect of hederagenin, cholesterol, and cholic acid derivatives on protozoal activity was measured *in vitro* as the breakdown of [^14^C] labeled bacteria by rumen protozoa as described by Wallace and McPherson (1987). Isotope-labeled bacteria were obtained by growing *Streptococcus bovis* in Wallace and McPherson media (Wallace and McPherson, 1987) containing [^14^C] leucine (1.89 μCi/7.5 mL tube) as the sole nitrogen source, for 24 h. Cultures were centrifuged (3,000 × *g* for 15 min), supernatant discarded and pellets re-suspended in 7 mL of simplex type salt solution (STS; Williams and Coleman, 1992) containing non-labeled leucine (^12^C-leucine, 5 mM). This process was repeated three times to prevent re-incorporation of released [^14^C] leucine by bacteria. The labeled bacterial suspension was sampled to determine its radioactivity and then it was used as the substrate in the incubations with rumen fluid.

Rumen digesta was obtained from four rumen-cannulated Holstein-Frisian cows (four replicates), fed at maintenance level (diet composed of perennial ryegrass hay and concentrate at 67:33 on DM basis). Animal procedures were carried out in accordance with the Animal Scientific Procedures Act 1986 and protocols were approved by the Aberystwyth University Ethical Committee. Rumen digesta was obtained before the morning feeding and strained through two layers of muslin and diluted with STS (1:1). Diluted rumen fluid (7.5 mL) was then incubated with labeled bacteria (0.5 mL) in tubes containing no additive (control) or 0.05, 0.1, 0.5, or 1 g/L of the modified triterpenes or steroids; hederoside B, a natural saponin isolated from ivy fruit, was also incubated at 0.05, 0.1, 0.5, and 1 g/L. Hederagenin *bis*-sulfate disodium salt (TSB38), cholesteryl succinate (TSB39), and lithocholic acid succinate (TSB42) were dissolved in dimethyl sulfoxide (DMSO) at 1% of the incubation volume. The rest of the derivatives and Hederoside B were solubilized in ethanol at 1% of the incubation volume as it has been shown that such concentration of ethanol in rumen fluid should not impair fermentation (Morgavi et al., 2004; Wallace et al., 2007). Two control treatments with 1% of either DMSO or ethanol were also included in the experimental design. Incubations were carried out at 39°C under a stream of CO_2_ and tubes were sampled at time 0 and at 1 h intervals up to 5 h using a syringe with a 19 gauge needle. Samples (0.5 mL) were acidified [by adding 125 μL of 25% (wt/vol) trichloroacetic acid and centrifuged (13,000 × *g* for 5 min). Supernatant (200 μL), was diluted with 2 mL of scintillation fluid to determine the radioactivity released by liquid-scintillation spectrometry (Hidex 300 SL, Lablogic Systems Ltd, Broomhill, UK]. Bacterial breakdown at each incubation time was expressed as the percentage of the acid-soluble radioactivity released relative to the total radioactivity present in the initial labeled bacteria (Wallace and McPherson, 1987).

### *In vitro* Batch Cultures

The initial protozoal population in the inoculum used in the incubations was quantified by optical microscope using the procedure described by Dehority (1993) and adapted by de la Fuente et al. (2006). Within the total population (5.34 log cells/mL), 65% were *Entodinium*, 8% Epidinium, 21% *Diplodinium*, 3% *Isotricha*, and 3% *Dasytricha*.

To estimate the stability of the antiprotozoal effect and measure the influence of the modified triterpene and steroids on fermentation parameters, strained rumen fluid from each cow was diluted 1:2 in artificial saliva solution (Menke and Steingass, 1988). Aliquots (30 mL) of the diluted strained rumen fluid were added anaerobically to 120 mL serum bottles (Sigma-Aldrich Ltd, Dorset, UK) containing 0.3 g of diet composed of ryegrass hay and barley (40:60), previously ground to pass through a 1-mm^2^ mesh screen. Treatments consisted of control incubations (0.3 g of diet only), with either ethanol or DMSO added at 1%, and incubations with the synthesized compounds (diluted in ethanol or DMSO, as previously described) at 0.5 or 1 g/L of the incubation. To compare the antiprotozoal effect of the synthesized compounds against that of a natural saponin from ivy, hederoside B (dissolved in ethanol) was incubated at 1 g/L. Bottles were incubated at 39°C under CO_2_ receiving a gentle mix before every sampling time. Samples at different time points (0, 4, 8, and 24 h) were collected for visual assessment of protozoa motility. Ciliate protozoa motility was assessed in 30 μL of sample against a common scale when examined at low magnification (100×) using light microscopy. This evaluation was conducted in less than 1 min/sample to avoid the cell damage originated by the oxygen and temperature exposure. A score between 0 (no whole protozoa evident) and 5 (all genera active) was given according to the scale described by Newbold (2010). Fermentation pattern, in terms of pH and VFA was determined after 24 h of the incubation. A subsample (4 mL) was diluted with 1 mL of deproteinizing solution (200 mL/L orthophosphoric acid containing 20 mmol/L of 2-ethylbutyric acid as an internal standard) for the determination of VFA using gas chromatography (Stewart and Duncan, 1985).

### Calculations and Statistical Analysis

A simple linear regression was conducted to model the relationship between the percentage of radioactivity released (relative to the ^14^C-bacterial inoculum) and the time (from 0 h to 5 h), as well as its correlation coefficient. The slope of this trend-line indicated the bacterial degradation rate (as % h^-1^) by the rumen protozoa and ultimately their activity. Trend line slopes as well as fermentation parameters were analyzed statistically by randomized block ANOVA, with individual cows as a blocking term. Inhibition of protozoa activity (% with respect to the control) was analyzed using ANOVA with treatment, dose and their interaction as fixed effects and cow as blocking term. When significant effects were detected across the different doses, means were compared by Fisher’s unprotected LSD test.

Protozoal motility was analyzed as a Repeated Measures Design, with treatment as main factor and incubation time as subject factor. A stability index, to estimate the persistence of the saponin effect over time, was calculated as the percentage of the motility at 8 h that remained at 24 h. Interaction between treatment and time as a measure of differential temporal dynamics between treatments was also considered. Differences were declared significant at *P* < 0.05 and considered as tendencies toward significance at *P* < 0.10. Genstat 15th Edition (VSN International, Hemel Hempstead, UK) was used.

## Results

### Acute Anti-protozoal Activity

The amount of bacteria degraded by protozoa increased linearly (*R*^2^ > 0.99) over the 5 h of incubation with both control treatments (with ethanol or with DMSO). For each derivative, the rate of bacterial degradation at different doses as compared with the control is shown in Supplemental Table S1. The inhibition of protozoa activity (**Table 1**) was significantly different between compounds and doses (*P* < 0.001). Derivatives TSB44, TSB45, TSB46, TSB47, TSB52, and TSB42 were more effective in inhibiting protozoa activity than hederoside B, the major ivy saponin. Among the cholesterol and cholic acid derivatives, TSB39, TSB40, and TSB43 were less effective against protozoa than the natural saponin (*P* < 0.001).

**Table 1:** Inhibition of protozoa activity (% in respect to the control, no addition) by hederagenin and bile acid derivatives, added at 0.05, 0.1, 0.5, or 1 g/L.

	Dose (g/L)			
	0.05	0.1	0.5	1
Hederoside B	5.11	22.0	86.0	84.6
**Hederagenin derivatives**				
TSB24: Hederagenin *bis*-succinate	5.72	18.8	96.5	100
TSB33: Hederagenin *bis*-(methylethylenglycolacetate)	13.6	29.7	51.3	64.5
TSB34: Hederagenin *bis*-(MeO-PEG4-carbonate)	7.69	14.1	65.5	69.6
TSB35: Hederagenin *bis*-glutarate	7.69	36.0	95.5	93.1
TSB36: Hederagenin *bis*-glycincarbamate	0.55	6.19	55.3	93.8
TSB37: Hederagenin *bis*-betainate dichloride	16.9	29.1	90.9	94.2
TSB38: Hederagenin *bis*-sulfate disodium salt	1.32	4.07	47.9	83.9
TSB44: Hederagenin *bis*-lactate	39.1	86.5	98.3	98.4
TSB45: Hederagenin *bis*-(2,2-dimethylsuccinate)	63.1	93.6	96.9	97.8
TSB46: Hederagenin bis-(3,3-dimethylglutarate)	75.3	93.0	97.2	96.7
TSB47: Hederagenin *bis*-adipate	29.6	78.1	98.0	94.0
TSB50: Hederagenin-*bis*-(diglycolate)	1.06	8.45	75.3	74.3
TSB51: Hederagenin *bis*-(diglycinate)	1.74	0.29	54.2	63.8
TSB52: Hederagenin *bis*-(3,3-dimethylsuccinate)	66.7	95.2	98.8	98.4
TSB58: Hederagenin *bis*-L-tartrate monomethyl ester	0	4.1	95.2	98.2
**Cholesterol and Cholic acid derivatives**				
TSB39: Cholesteryl succinate	6.42	18.2	17.7	17.6
TSB40: Cholic succinate	25.2	23.5	26.5	42.6
TSB41: Cholic tri-succinate	26.4	21.9	32.9	67.1
TSB42: Lithocholic succinate	75.1	92.8	97.5	97.4
TSB43: Chenodesoxycholic *bis*-succinate	1.68	5.66	15.5	53.4
SED Treatment	4.94^∗∗∗^			
Dose	2.16^∗∗∗^			
Treatment × Dose	9.88^∗∗∗^			

*SED, Standard error of the difference; ^∗∗∗^*P* < 0.001*.

### Stability of the Antiprotozoal Effect and Effect on Fermentation Parameters

Based on the observed effects of the synthesized compounds on bacterial breakdown by protozoa, the two highest doses of these derivatives (0.5 and 1 g/L) and hederoside B at 1 g/L, were tested over 24 h in *in vitro* incubations. Protozoa motility over time was assessed and fermentation parameters were determined after 24 h of incubation. Due to the number of compounds tested, the experiment was carried out in different batches and hence the slightly different values for fermentation parameters between control incubations. To overcome this issue, we have compared the effects of each compound against the control run with the same batch of rumen fluid.

**FIGURE 3:**
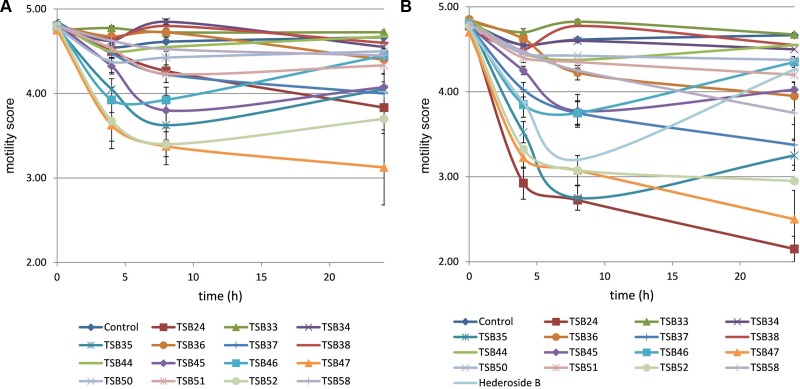
**Protozoa motility over 24 h in the absence (control) or presence of different hederagenin derivatives at 0.5 (A)** and 1 g/L **(B)**. Hederoside B was used as a positive control at 1 g/L. Error bars indicate the standard error of the difference for each time point (*n* = 4).

**FIGURE 4:**
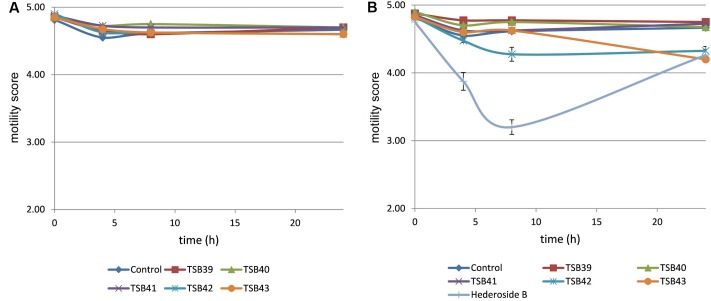
**Protozoa motility over 24 h in the absence (control) or presence of different cholesterol and cholic acid derivatives at 0.5 (A)** and 1 g/L **(B)**. Hederoside B was used as a positive control at 1 g/L. Error bars indicate the standard error of the difference for each time point (*n* = 4).

**FIGURE 5:**
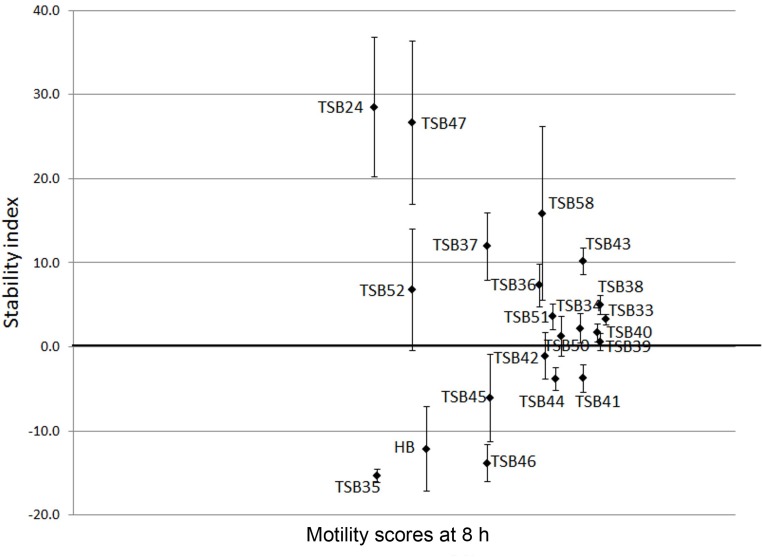
**Stability index (calculated as the percentage of the motility at 8 h that remained at 24 h) against motility scores at 8 h in the presence of hederagenin and cholesterol and cholic acid derivatives and hederoside B (HB) at 1 g/L**. Error bars indicate the standard error of the difference (*n* = 4).

Cell motility, measured as an index of protozoa viability, remained unaltered (score of 4.8) over the 24 h incubation period in control incubations with ethanol or DMSO (**Figures 3**, **4**). The effect of hederagenin derivatives when added at 0.5 g/L or 1 g/L is shown in **Figures 3A,B**, respectively. Although, 1 g/L of hederoside B decreased protozoa motility at 4 and 8 h of the incubation (with scores of 3.88 and 3.20, respectively), there was a strong treatment × time interaction (*P* = 0.05), and protozoal motility recovered afterward (reaching a score of 4.26 at 24 h), suggesting the expected degradation of the saponin during the incubation. Some of the derivatives, TSB45 and TSB46, showed the same effect as the natural saponin, initially decreasing protozoa motility but with motility recovering after 24 h (treatment × time interaction, *P* < 0.05). Other derivatives, TSB24, TSB47, and TSB52, added at 1 g/L, however, resulted in a greater decrease in protozoa activity over time (*P* < 0.001; scores of around 3; no motility or activity evident) with no sign of recovery in motility. Indeed, vacuoles were visible at 24 h suggesting protozoal death (scores of 2.15–2.9). Only few of the hederagenin derivatives (TSB33, TSB34, TSB38, and TSB44) did not show an effect on protozoa motility (*P* > 0.05) at any of the concentrations tested. Cholesterol and cholic acid derivatives did not seem to be effective in reducing protozoa motility over time as shown in **Figure 4**. Only TSB42 when added at 1 g/L showed a slight decrease in protozoa motility after 8 and 24 h of incubation (treatment x time interaction, *P* = 0.017; **Figure 4B**). A stability index, to estimate the persistence of the saponin effect over time, was calculated as the percentage of the motility at 8 h that remained at 24 h (**Figure 5**). Whereas the compounds located above the origin on the *y*-axis were stable (persistent effect on protozoal motility at 24 h; e.g., TS24, TSB37, TSB47), those below the origin on the y-axis showed a loss of effect on protozoal motility (recovery of motility after 24 h; e.g., TSB35, TSB46, hederoside B). The derivatives close to or on the origin of the *y*-axis (e.g., TSB50, TSB51) correspond to those compounds that were less effective against protozoa (scores of about 4.5 at 8 h) but with an effect that was maintained at 24 h.

Neither the natural saponin, hederoside B, nor the modified triterpenes or steroids caused a decrease in pH (*P* > 0.05; **Table 2**); indeed, pH was slightly greater in the presence of TSB35 and TSB37 at 0.5 and 1 g/L (*P* < 0.001) in comparison to the control. Similarly, no effect on the concentration of total VFA was observed in incubations with hederoside B or with most of the derivatives (*P* > 0.05; **Table 3**). Only TSB35 and TSB36 caused a reduction in the concentration of VFA (*P* < 0.05) when added at 0.5 and 1 g/L. Also, some of the derivatives decreased the molar proportion of acetate (**Table 4**; *P* < 0.05). Almost all treatments caused shifts in the molar proportions of VFA toward lower butyrate and higher propionate (*P* < 0.05), to different extents depending on the compound (**Tables 5**, **6**).

**Table 2:** Effect of Hederagenin and bile acid derivatives, added at 0.5 or 1 g/L, on pH after 24 h of incubation (batch culture).

	Dose g/L				
	0	0.5	1	SED	*P*
		pH			
Hederoside B	6.03	–	6.09	0.048	0.253
**Hederagenin derivatives**					
TSB24: Hederagenin *bis*-succinate	6.16	6.11	6.15	0.039	0.493
TSB33: Hederagenin *bis*- (methylethylenglycolacetate)	6.31	6.32	6.31	0.009	0.824
TSB34: Hederagenin *bis*-(MeO-PEG4-carbonate)	6.31	6.31	6.31	0.013	0.924
TSB35: Hederagenin *bis*-glutarate	6.31^a^	6.38^b^	6.38^b^	0.019	0.017
TSB36: Hederagenin *bis*-glycincarbamate	6.31	6.32	6.29	0.019	0.39
TSB37: Hederagenin *bis*-betainate dichloride	6.31^a^	6.36^b^	6.39^c^	0.008	<0.001
TSB38: Hederagenin *bis*-sulfate disodiumsalt	6.41	6.39	6.40	0.014	0.385
TSB44: Hederagenin *bis*-lactate	6.03	6.04	6.04	0.031	0.874
TSB45: Hederagenin *bis*-(2,2-dimethylsuccinate)	6.03^a^	6.12^b^	6.12^b^	0.033	0.051
TSB46: Hederagenin *bis*-(3,3-dimethylglutarate)	6.16	6.11	6.10	0.034	0.32
TSB47: Hederagenin *bis*-adipate	6.16	6.11	6.15	0.043	0.567
TSB50: Hederagenin *bis*-(diglycolate)	6.16	6.10	6.11	0.036	0.294
TSB51: Hederagenin *bis*-(diglycinate)	6.16	6.06	6.10	0.038	0.122
TSB52: Hederagenin *bis*-(3,3-dimethylsuccinate)	6.16	6.14	6.16	0.042	0.834
TSB58: Hederagenin *bis*-L-tartrate monomethyl ester	6.16	6.12	6.12	0.034	0.464
**Cholesterol and Cholic acid derivatives**					
TSB39: Cholesteryl succinate	6.18	6.20	6.18	0.031	0.74
TSB40: Cholic succinate	6.03	6.06	6.06	0.026	0.419
TSB41: Cholic tri-succinate	6.03	6.04	6.00	0.019	0.263
TSB42: Lithocholic succinate	6.18	6.19	6.18	0.020	0.876
TSB43: Chenodesoxycholic *bis*-succinate	6.03	6.05	6.03	0.021	0.508

^a-c^*Means with different superscript differ (*n* = 4)*.

**Table 3:** Effect of Hederagenin and bile acid derivatives, added at 0.5 or 1 g/L, on total VFA (mM) after 24 h of incubation (batch culture).

	Dose g/L				
	0	0.5	1	SED	*P*
	total VFA (mM)				
Hederoside B	82.5	–	77.2	3.1	0.185
**Hederagenin derivatives**					
TSB24: Hederagenin *bis*-succinate	70.1	65.4	70.9	4.39	0.448
TSB33: Hederagenin *bis*-(methylethylenglycolacetate)	80.1	78.6	76.3	3.88	0.647
TSB34: Hederagenin *bis*-(MeO-PEG4-carbonate)	80.1	75.0	73.9	4.19	0.355
TSB35: Hederagenin *bis*-glutarate	80.1^b^	68.7^a^	66.7^a^	3.14	0.011
TSB36: Hederagenin *bis*-glycincarbamate	80.1^b^	71.2^a^	69.6^a^	3.42	0.045
TSB37: Hederagenin *bis*-betainate dichloride	80.1	70.8	72.1	3.34	0.065
TSB38: Hederagenin *bis*-sulfate disodiumsalt	74.3	70.6	67.3	6.32	0.568
TSB44: Hederagenin *bis*-lactate	82.5	82.0	82.0	2.04	0.958
TSB45: Hederagenin *bis*-(2,2-dimethylsuccinate)	82.5	80.9	73.4	3.47	0.079
TSB46: Hederagenin *bis*-(3,3-dimethylglutarate)	70.1	73.8	73.8	3.29	0.467
TSB47: Hederagenin *bis*-adipate	70.1	73.1	70.8	3.16	0.620
TSB50: Hederagenin *bis*-(diglycolate)	70.1	73.3	73.3	3.84	0.638
TSB51: Hederagenin *bis*-(diglycinate)	70.1	69.7	74.0	3.84	0.503
TSB52: Hederagenin *bis*-(3,3-dimethylsuccinate)	70.1	69.5	71.3	4.49	0.914
TSB58: Hederagenin *bis*-L-tartrate monomethyl ester	70.1	71.9	72.3	3.64	0.811
**Cholesterol and Cholic acid derivatives**					
TSB39: Cholesteryl succinate	72.5	72.5	73.3	0.579	0.333
TSB40: Cholic succinate	82.5	80.4	81.3	2.48	0.704
TSB41: Cholic tri-succinate	82.5	81.5	79.1	2.85	0.512
TSB42: Lithocholic succinate	72.5	73.0	66.5	2.46	0.070
TSB43: Chenodesoxycholic *bis*-succinate	82.5	78.9	77.3	2.98	0.279

^a,b^*Means with different superscript differ (*n* = 4)*.

**Table 4:** Effect of Hederagenin and bile acid derivatives, added at 0.5 or 1 g/L, on Acetate (%) after 24 h of incubation (batch culture).

	Dose g/L				
	0	0.5	1	SED	*P*
	Acetate % of total VFA				
Hederoside B	64.8	–	59.8	0.681	0.005
**Hederagenin derivatives**					
TSB24: Hederagenin *bis*-succinate	64.1^b^	62.1^b^	59.4^a^	0.837	0.004
TSB33: Hederagenin *bis*- (methylethylenglycolacetate)	66.1	65.3	65.2	0.435	0.148
TSB34: Hederagenin *bis*-(MeO-PEG4-carbonate)	66.1	65.4	65.2	0.575	0.31
TSB35: Hederagenin *bis*-glutarate	66.1^c^	60.2^b^	57.2^a^	0.468	<0.001
TSB36: Hederagenin *bis*-glycincarbamate	66.1^b^	64.7^a^	64.1^a^	0.472	0.012
TSB37: Hederagenin *bis*-betainate dichloride	66.1^c^	65.5^b^	58.3^a^	0.751	<0.001
TSB38: Hederagenin *bis*-sulfate disodiumsalt	62.3	62.7	61.8	0.502	0.259
TSB44: Hederagenin *bis*-lactate	64.8	65.4	65.3	0.964	0.787
TSB45: Hederagenin *bis*-(2,2-dimethylsuccinate)	64.8^b^	61.7^a^	61.1^a^	1.177	0.041
TSB46: Hederagenin *bis*-(3,3-dimethylglutarate)	64.1^b^	61.6^a^	60.3^a^	0.767	0.007
TSB47: Hederagenin *bis*-adipate	64.1^c^	59.1^b^	56.8^a^	0.875	<0.001
TSB50: Hederagenin *bis*-(diglycolate)	64.1	64.4	65.2	1.071	0.582
TSB51: Hederagenin *bis*-(diglycinate)	64.1	65.6	64.7	0.77	0.207
TSB52: Hederagenin *bis*-(3,3-dimethylsuccinate)	64.1^b^	60.8^a^	60.3^a^	0.827	0.008
TSB58: Hederagenin *bis*-L-tartrate monomethyl ester	64.1	65.5	65.3	0.841	0.244
**Cholesterol and cholic acid derivatives**					
TSB39: Cholesteryl succinate	61.8	61.8	61.8	0.19	0.993
TSB40: Cholic succinate	64.8	65.0	64.7	0.941	0.948
TSB41: Cholic tri-succinate	64.8	65.5	64.9	1.02	0.744
TSB42: Lithocholic succinate	61.8^b^	61.2^b^	60.1^a^	0.322	0.005
TSB43: Chenodesoxycholic *bis*-succinate	64.8^b^	64.4^b^	61.1^a^	1.25	0.047

^a-c^*Means with different superscript differ (*n* = 4)*.

**Table 5:** Effect of Hederagenin and bile acid derivatives, added at 0.5 or 1 g/L, on Propionate (%) after 24 h of incubation (batch culture).

	Dose g/L				
	0	0.5	1	SED	*P*
	Propionate % of total VFA				
Hederoside B	20.1	–	27.2	1.04	0.006
**Hederagenin derivatives**					
TSB24: Hederagenin *bis*-succinate	18.3^a^	25.9^b^	30.5^c^	1.13	<0.001
TSB33: Hederagenin *bis*-(methylethylenglycolacetate)	18.6^a^	19.7^ab^	20.3^b^	0.516	0.038
TSB34: Hederagenin *bis*-(MeO-PEG4-carbonate)	18.6^a^	20.1^ab^	20.8^b^	0.715	0.05
TSB35: Hederagenin *bis*-glutarate	18.6^a^	28.0^b^	31.4^c^	0.781	<0.001
TSB36: Hederagenin *bis*-glycincarbamate	18.6^a^	20.7^b^	22.5^c^	0.683	0.004
TSB37: Hederagenin *bis*-betainate dichloride	18.6^a^	24.9^b^	30.6^c^	1.12	<0.001
TSB38: Hederagenin *bis*-sulfate disodiumsalt	20.9^a^	22.3^a^	24.5^b^	0.575	0.002
TSB44: Hederagenin *bis*-lactate	20.1	19.5	20.5	1.10	0.632
TSB45: Hederagenin *bis*-(2,2-dimethylsuccinate)	20.1^a^	27.2^b^	28.3^b^	1.44	0.002
TSB46: Hederagenin *bis*-(3,3-dimethylglutarate)	18.3^a^	27.4^b^	28.9^b^	0.984	<0.001
TSB47: Hederagenin *bis*-adipate	18.3^a^	30.4^b^	33.7^c^	1.00	<0.001
TSB50: Hederagenin *bis*-(diglycolate)	18.3^a^	20.2^b^	20.5^b^	0.698	0.041
TSB51: Hederagenin *bis*-(diglycinate)	18.3^a^	19.8^b^	22.4^c^	0.496	<0.001
TSB52: Hederagenin *bis*-(3,3-dimethylsuccinate)	18.3^a^	28.6^b^	29.7^b^	1.18	<0.001
TSB58: Hederagenin *bis*-L tartrate monomethyl ester	18.3^a^	19.0^a^	20.9^b^	0.579	0.011
**Cholesterol and Cholic acid derivatives**					
TSB39: Cholesteryl succinate	21.0	20.8	21.0	0.167	0.458
TSB40: Cholic succinate	20.1	19.7	20.5	1.139	0.817
TSB41: Cholic tri-succinate	20.1	19.1	19.6	0.961	0.643
TSB42: Lithocholic succinate	21.0^a^	22.8^b^	25.0^c^	0.559	0.001
TSB43: Chenodesoxycholic *bis*-succinate	20.1	20.6	23.9	1.476	0.079

^a-c^*Means with different superscript differ (*n* = 4)*.

**Table 6:** Effect of Hederagenin and bile acid derivatives, added at 0.5 or 1 g/L, on Butyrate (%) after 24 h of incubation (batch culture).

	Dose g/L				
	0	0.5	1	SED	*P*
	Butyrate % of total VFA				
Hederoside B	12.1	–	9.83	0.427	0.013
**Hederagenin derivatives**					
TSB24: Hederagenin *bis*-succinate	14.3	8.8	7.2	0.606	<0.001
TSB33: Hederagenin *bis*-(methylethylenglycolacetate)	11.7	11.5	11.1	0.25	0.1
TSB34: Hederagenin *bis*-(MeO-PEG4-carbonate)	11.7^b^	11.2^ab^	10.7^a^	0.231	0.017
TSB35: Hederagenin *bis*-glutarate	11.7^b^	7.92^a^	7.54^a^	0.253	<0.001
TSB36: Hederagenin *bis*-glycincarbamate	11.7^c^	11.2^b^	10.1^a^	0.150	<0.001
TSB37: Hederagenin *bis*-betainate dichloride	11.7^c^	9.17^b^	7.70^a^	0.375	<0.001
TSB38: Hederagenin *bis*-sulfate disodiumsalt	12.8^c^	11.3^b^	10.2^a^	0.4	0.002
TSB44: Hederagenin *bis*-lactate	12.1^b^	11.7^b^	11.0^a^	0.173	0.003
TSB45: Hederagenin *bis*-(2,2-dimethylsuccinate)	12.1^b^	7.74^a^	7.62^a^	0.394	<0.001
TSB46: Hederagenin *bis*-(3,3-dimethylglutarate)	14.3^b^	8.26^a^	7.76^a^	0.571	<0.001
TSB47: Hederagenin *bis*-adipate	14.3^b^	7.35^a^	6.78^a^	0.608	<0.001
TSB50: Hederagenin *bis*-(diglycolate)	14.3^b^	12.6^a^	11.7^a^	0.506	0.005
TSB51: Hederagenin *bis*-(diglycinate)	14.3^b^	11.7^a^	10.2^a^	0.606	0.001
TSB52: Hederagenin *bis*-(3,3-dimethylsuccinate)	14.3^b^	7.86^a^	7.33^a^	0.746	0.001
TSB58: Hederagenin *bis*-L-tartrate monomethyl ester	14.3^c^	12.5^b^	11.2^a^	0.383	<0.001
**Cholesterol and Cholic acid derivatives**					
TSB39: Cholesteryl succinate	12.9	13.0	12.8	0.094	0.341
TSB40: Cholic succinate	12.1^b^	11.8^b^	11.3^a^	0.178	0.013
TSB41: Cholic tri-succinate	12.1	12.0	12.1	0.322	0.938
TSB42: Lithocholic succinate	12.9^c^	11.4^b^	10.2^a^	0.257	<0.001
TSB43: Chenodesoxycholic *bis*-succinate	12.1^c^	11.5^b^	10.9^a^	0.176	0.002

^a-c^Means with different superscript differ (*n* = 4).

The natural saponin, hederoside B, decreased acetate and butyrate molar proportions by 8 and 18%, respectively, whereas it increased that of propionate by 35%, in comparison to the control. The greatest effect was observed with TSB35 (hederagenin *bis*-glutarate), TSB37 (hederagenin *bis*-betainate dichloride) and TSB47 (hederagenin *bis*-apidate) which, when added at 1 g/L, decreased the molar proportion of acetate and butyrate by 11–13.5% and 35.5–52.7%, respectively, with an increase in propionate of 64.5–84.2%. Cholesteryl succinate (TSB39) and cholic tri-succinate (TSB41) did not have any effect on the molar proportions of VFA. Cholic succinate (TSB40) caused only a slight decrease in butyrate (*P* = 0.013) at 1 g/L, as compared to the control. TSB42 and TSB43 also resulted in decreases in acetate and butyrate and increases in propionate although to a lesser extent than those caused by hederoside B. Molar proportions of branched-chain VFA (BCVFA, i.e., isobutyrate and isovalerate) decreased (*P* < 0.05) in incubations with TSB24 (–13%) and TSB38 (-16%) at 1 g/L and TSB50, TSB51, TSB52, and TSB58 at 0.5 and 1 g/L (decreases of 22–24% at 1 g/L; **Table 7**). TSB43, however, resulted in an increase (*P* = 0.044) in BCVFA when added at 1 g/L (+54%; **Table 7**). This was mainly due to changes in isovalerate rather than isobutyrate (Supplemental Tables S2, S3).

**Table 7:** Effect of Hederagenin and bile acid derivatives, added at 0.5 or 1 g/L, on branched chain volatile fatty acids (BCVFA) (%) after 24 h of incubation (batch culture).

	Dose g/L				
	0	0.5	1	SED	*P*
	BCVFA % of total VFA				
Hederoside B	1.95	–	2.08	0.161	0.474
**Hederagenin derivatives**					
TSB24: Hederagenin *bis*-succinate	2.08^b^	1.96^ab^	1.81^a^	0.081	0.045
TSB33: Hederagenin *bis*-(methylethylenglycolacetate)	2.47	2.29	2.29	0.126	0.307
TSB34: Hederagenin *bis*-(MeO-PEG4-carbonate)	2.47^a^	2.19^ab^	2.11^b^	0.124	0.056
TSB35: Hederagenin *bis*-glutarate	2.47	2.23	2.65	0.216	0.219
TSB36: Hederagenin *bis*-glycincarbamate	2.47	2.33	2.34	0.229	0.775
TSB37: Hederagenin *bis*-betainate dichloride	2.47	2.26	2.35	0.082	0.103
TSB38: Hederagenin *bis*-sulfate disodiumsalt	2.64^b^	2.42^ab^	2.22^a^	0.120	0.032
TSB44: Hederagenin *bis*-lactate	1.95	2.30	2.07	0.275	0.469
TSB45: Hederagenin *bis*-(2,2-dimethylsuccinate)	1.95	2.36	1.85	0.321	0.305
TSB46: Hederagenin *bis*-(3,3-dimethylglutarate)	2.08^b^	1.68^a^	2.01^b^	0.096	0.012
TSB47: Hederagenin *bis*-adipate	2.08	1.91	1.77	0.166	0.263
TSB50: Hederagenin *bis*-(diglycolate)	2.08^b^	1.79^a^	1.62^a^	0.11	0.016
TSB51: Hederagenin *bis*-(diglycinate)	2.08^b^	1.69^a^	1.63^a^	0.08	0.003
TSB52: Hederagenin *bis*-(3,3-dimethylsuccinate)	2.08^b^	1.59^a^	1.58^a^	0.088	0.002
TSB58: Hederagenin *bis*-L-tartrate monomethyl ester	2.08^b^	1.86^b^	1.57^a^	0.091	0.004
**Cholesterol and Cholic acid derivatives**					
TSB39: Cholesteryl succinate	3.10	3.23	3.13	0.261	0.879
TSB40: Cholic succinate	1.95	2.40	2.45	0.209	0.1
TSB41: Cholic tri-succinate	1.95	2.30	2.31	0.1772	0.141
TSB42: Lithocholic succinate	3.10	3.63	3.49	0.191	0.203
TSB43: Chenodesoxycholic *bis*-succinate	1.95^a^	2.52^ab^	3.00^b^	0.319	0.044

^a-b^*Means with different superscript differ (*n* = 4)*.

## Discussion

The biological activity of saponins depends not only on the type of aglycone but also on the sugar composition and arrangement (Wina et al., 2006). The haemolytic action of saponins is believed to be the result of the affinity of the aglycone moiety for membrane sterols, particularly cholesterol with which they form insoluble complexes. It has been shown that monodesmosidic saponins (a single sugar chain) were generally more active than bidesmosidic ones (two sugar chains) (Voutquenne et al., 2002). A further study (Chwalek et al., 2006) testing different hederagenin diglycosides concluded that even the substitution of a monosaccharide with another monosaccharide within the sugar chain may change biological activity of saponins. As far as we know, no studies on the correlation between the haemolytic activity and antiprotozoal activity or on the relationship between saponin structure and antiprotozoal activity in the rumen have been carried out.

Although the antiprotozoal effect of saponins has been consistently shown in *in vitro* studies (Wina et al., 2005), it was also found to be transient (Newbold et al., 1997; Teferedegne et al., 1999). This transient nature has been associated to the degradation of saponins, i.e., the cleavage of the glycosidic bonds toward the aglycone leaving the inactive sapogenin behind, by rumen bacteria rather than to the ability of rumen protozoa to become resistant (Newbold et al., 1997). Makkar and Becker (1997) reported the disappearance of saponins from quillaja over time when incubated with buffered rumen fluid, with a reduction of 50% after 12 h and by 100% at 24 h of the incubation. In the present study, we hypothesized that the substitution of the sugar moiety of the saponin with small polar residues would produce sapogen-like analogs that might be resistant to ruminal degradation. Both the acute antiprotozoal activity and the stability of that effect over 24 h of fifteen hederagenin *bis*-esters esterified with two identical groups (**Figure 1**), and five cholesterol and cholic acid based derivatives carrying one to three succinate residues (**Figure 2**) was evaluated. Our 5 h *in vitro* incubations results showed that, irrespective of their resistance to degradation, some of the hederagenin derivatives were more effective in reducing protozoa activity than the natural saponin hederoside B. The greatest effect was shown with TSB45, TSB46, and TSB52 which reduced protozoa activity by 63–75% when they were incubated at 0.05 g/L. Interestingly among the cholesterol and cholic acid derivatives, TSB39 (cholesteryl succinate) had the lowest antiprotozoal effect and, TSB42 (lithocholic acid succinate) was one of the most effective compounds tested, decreasing protozoa activity by 75% when added at 0.05 g/L. These results agree with the observations of Takechi et al. (1996), who showed that the biological activity that a specific chemical residue may provide is not transferable from one derivative to another. To study if the synthesized derivatives were still effective against protozoa over a longer period of time, *in vitro* incubations were carried out sampling at 0, 4, 8, and 24 h to assess the stability of the derivatives in a mixed rumen population. Derivatives TSB24, TSB47, and TSB52 seemed to be very effective in causing a decrease in protozoa motility over time without recovery after 24 h, contrary to the results observed for hederoside B and the rest of compounds. Surprisingly, none of the cholesterol and cholic acid derivatives showed an effect on protozoa motility. Although TSB42 had a strong effect in bacterial breakdown by protozoa over 5 h of incubation, little effect on protozoa motility was observed in 24 h *in vitro* batch cultures. These results may suggest a quicker degradation, and thus the loss of activity, of this compound by rumen bacteria as compared with other derivatives tested. It is apparent that the compounds that showed a high level of acute toxicity against protozoa were not always the most stable ones over time. A stability index was calculated as the percentage of the 8 h activity that remained after 24 h (**Figure 5**). Even though TSB35 reduced protozoa activity by 93% when added at 1 g/L, this compound was among the least stable derivatives. TSB24 and TSB47, however, showed both high toxicity (reduction of protozoa activity of 95–100%) and stability over time.

Most of the hederagenin derivatives did not influence total VFA concentration. However, shifts in the molar proportions of VFA toward lower acetate and butyrate which was compensated by a higher propionate were observed. These changes have been previously reported when using different sources of saponins (Wina et al., 2005; Patra and Saxena, 2009a; Jayanegara et al., 2014). The shifts in the molar proportions of butyrate and propionate shown in the presence of TSB35, TSB37, and TSB47 were, however, much greater than those that would have been expected because of defaunation. A recent meta-analysis showed that defaunation decreased butyrate by 22% with no effect on propionate (Newbold et al., 2015). It should be pointed out that TSB37 was of low purity (66%) and thus, this hederagenin derivative could have been more effective than others with higher purity. However, it is possible that the effects observed in the presence of TSB37 were due to the impurities in this derivative. Although our target in using the synthesized compounds was to control protozoal activity, other microorganisms may also have been directly or indirectly affected by the derivatives resulting in further effects on rumen fermentation. Indeed, a direct effect of saponins on bacteria, probably mediated by disruption of the cell membrane (Patra and Saxena, 2009a,b; Bodas et al., 2012), has been reported. Similarly, saponins can exert antifungal activity by the interaction with membrane sterols leading to pore formation and loss of membrane integrity (Goel et al., 2008; Patra and Saxena, 2009a,b).

Clearly modifications in the structure of hederagenin resulted in compounds with different biological activities *in vitro*. Whereas some compounds (TSB24) were more effective in reducing protozoa activity and motility, others (TSB37) caused a substantial increase in propionate. If the effect of these compounds can be confirmed *in vivo*, the use of these modified triterpenes in ruminant nutrition will have the potential to improve the efficiency of nitrogen utilization and decrease methane production thus potential boosting productivity.

## Conclusion

Most of the hederagenin *bis*-esters, and in particular hederagenin *bis*-succinate (TSB24), hederagenin *bis*-betainate dichloride (TSB37), and hederagenin *bis*-adipate (TSB47) had a persistent effect against rumen protozoa *in vitro*, shifting the fermentation pattern toward higher propionate and lower butyrate. The confirmation of these effects *in vivo* would help to determine if these novel chemically modified triterpenes could potentially be used in ruminant diets as an effective defaunation agent to, ultimately, increase nitrogen utilization, decrease methane emissions, and enhance animal production.

## Author Contributions

ER-M, SD, CW, MB, ML, DP, RB, and CN contributed to the conception and design of the work; ER-M and GdF conducted the research; ER-M wrote the manuscript; ER-M, GdF, SD, CW, MB, ML, DP, RB, and CN reviewed the manuscript. ER-M and CN had primary responsibility for the final content. All authors read and approved the final manuscript.

## Conflict of Interest Statement

The authors declare that the research was conducted in the absence of any commercial or financial relationships that could be construed as a potential conflict of interest.
