# Supplementary material for: Antiprotozoal Effect of Saponins in the Rumen Can Be Enhanced by Chemical Modifications in Their Structure
Source: Front Microbiol. 2017 Mar 16;8:399. doi: 10.3389/fmicb.2017.00399 (PMC5361656; doi:10.3389/fmicb.2017.00399)
Supplement: Supplementary file 1 [file Table_1.DOCX]

Table S1. Effect of Hederagenin and bile acid derivatives, added at 0.05, 0.1, 0.5 or 1 g/L, on rumen protozoal activity assessed *in vitro* as the amount of 14C-labeled bacteria broken down by rumen protozoa (% of the initial radioactivity released per hour).

|  | Dose (g/L) | | | | |  |  |
| --- | --- | --- | --- | --- | --- | --- | --- |
|  | 0 | 0.05 | 0.1 | 0.5 | 1 | SED | P |
|  | Protozoal activity (% bacterial breakdown/h) | | | | |  |  |
| Hederoside B | 3.10^b^ | 3.01^b^ | 2.44^b^ | 0.42^a^ | 0.47^a^ | 0.322 | <0.001 |
| **Hederagenin derivatives** |  |  |  |  |  |  |  |
| TSB24: Hederagenin *bis*-succinate | 4.36^c^ | 4.31^c^ | 3.59^b^ | 0.15^a^ | 0.00^a^ | 0.290 | <0.001 |
| TSB33: Hederagenin *bis*- (methylethylenglycolacetate) | 2.58^c^ | 2.41^c^ | 1.82^b^ | 1.31^a^ | 1.01^a^ | 0.187 | <0.001 |
| TSB34: Hederagenin *bis-*(MeO-PEG4-carbonate) | 2.58^b^ | 2.38^b^ | 2.26^b^ | 0.96^a^ | 0.83^a^ | 0.181 | <0.001 |
| TSB35: Hederagenin *bis*-glutarate | 2.58^b^ | 2.44^b^ | 1.77^b^ | 0.13^a^ | 0.18^a^ | 0.396 | <0.001 |
| TSB36: Hederagenin *bis*-glycincarbamate | 2.58^c^ | 2.88^c^ | 2.47^c^ | 1.16^b^ | 0.17^a^ | 0.276 | <0.001 |
| TSB37: Hederagenin *bis-*betainate dichloride | 2.58^b^ | 2.11^b^ | 1.88^b^ | 0.22^a^ | 0.17^a^ | 0.372 | <0.001 |
| TSB38: Hederagenin *bis*-sulfate disodiumsalt | 2.16^c^ | 2.38^c^ | 2.29^c^ | 1.05^b^ | 0.30^a^ | 0.29 | <0.001 |
| TSB44: Hederagenin *bis*-lactate | 7.15^c^ | 4.56^b^ | 1.05^a^ | 0.12^a^ | 0.11^a^ | 0.978 | <0.001 |
| TSB45: Hederagenin *bis*-(2,2-dimethylsuccinate) | 7.15^c^ | 2.46^b^ | 0.38^a^ | 0.19^a^ | 0.13^a^ | 0.829 | <0.001 |
| TSB46: Hederagenin *bis*-(3,3-dimethylglutarate) | 7.15^b^ | 1.86^a^ | 0.49^a^ | 0.19^a^ | 0.19^a^ | 0.812 | <0.001 |
| TSB47: Hederagenin *bis-*adipate | 7.15^c^ | 4.83^b^ | 1.49^a^ | 0.13^a^ | 0.39^a^ | 0.871 | <0.001 |
| TSB50: Hederagenin *bis*-(diglycolate) | 7.15^b^ | 7.52^b^ | 6.55^b^ | 1.74^a^ | 1.99^a^ | 0.604 | <0.001 |
| TSB51: Hederagenin *bis*-(diglycinate) | 7.15^b^ | 7.31^b^ | 7.20^b^ | 3.24^a^ | 2.44^a^ | 0.74 | <0.001 |
| TSB52: Hederagenin *bis*-(3,3-dimethylsuccinate) | 7.15^c^ | 2.45^b^ | 0.32^a^ | 0.09^a^ | 0.10^a^ | 0.813 | <0.001 |
| TSB58: Hederagenin *bis*-L-tartrate monomethyl ester | 3.10^b^ | 3.25^b^ | 3.22^b^ | 0.15^a^ | 0.05^a^ | 0.309 | <0.001 |
| **Cholesterol and Cholic acid derivatives** |  |  |  |  |  |  |  |
| TSB39: Cholesteryl succinate | 2.16 | 2.21 | 1.96 | 1.92 | 2.01 | 0.2 | 0.543 |
| TSB40: Cholic succinate | 2.58^b^ | 1.96^ab^ | 2.06^ab^ | 1.92^a^ | 1.50^a^ | 0.285 | 0.036 |
| TSB41: Cholic tri-succinate | 2.58^c^ | 1.86^b^ | 2.07^bc^ | 1.83^b^ | 0.94^a^ | 0.295 | 0.002 |
| TSB42: Lithocholic succinate | 2.98^b^ | 0.65^a^ | 0.20^a^ | 0.07^a^ | 0.07^a^ | 0.303 | <0.001 |
| TSB43: Chenodesoxycholic *bis*-succinate | 3.10^bc^ | 3.27^c^ | 3.03^bc^ | 2.61^b^ | 1.43^a^ | 0.255 | <0.001 |

^a–c^Means with different superscript differ (*n*=4).
