# Supplementary material for: Antiprotozoal Effect of Saponins in the Rumen Can Be Enhanced by Chemical Modifications in Their Structure
Source: Front Microbiol. 2017 Mar 16;8:399. doi: 10.3389/fmicb.2017.00399 (PMC5361656; doi:10.3389/fmicb.2017.00399)
Supplement: Supplementary file 2 [file Table_2.DOCX]

Table S2. Effect of hederagenin and bile acid derivatives, added at 0.5 or 1 g/L, on Isobutyrate (%) after 24 h of incubation (batch culture)

|  | Dose g/L | | |  |  |
| --- | --- | --- | --- | --- | --- |
|  | 0 | 0.5 | 1 | SED | P |
|  | Isobutyrate % of total VFA | | |  |  |
| Hederoside B | 1.07 | - | 1.24 | 0.083 | 0.135 |
|  |  |  |  |  |  |
| **Hederagenin derivatives** |  |  |  |  |  |
| TSB24: Hederagenin *bis*-succinate | 1.03 | 1.02 | 0.99 | 0.085 | 0.888 |
| TSB33: Hederagenin *bis*-(methylethylenglycolacetate) | 1.49 | 1.34 | 1.37 | 0.106 | 0.378 |
| TSB34: Hederagenin *bis*-(MeO-PEG4-carbonate) | 1.49 | 1.30 | 1.25 | 0.116 | 0.162 |
| TSB35: Hederagenin *bis*-glutarate | 1.49 | 1.32 | 1.68 | 0.202 | 0.284 |
| TSB36: Hederagenin *bis*-glycincarbamate | 1.49 | 1.41 | 1.45 | 0.196 | 0.915 |
| TSB37: Hederagenin *bis*-betainate dichloride | 1.49 | 1.39 | 1.48 | 0.074 | 0.397 |
| TSB38: Hederagenin *bis*-sulfate disodium salt | 1.53 | 1.43 | 1.28 | 0.104 | 0.130 |
| TSB44: Hederagenin *bis*-lactate | 1.07 | 1.37 | 1.26 | 0.217 | 0.439 |
| TSB45: Hederagenin *bis*-(2,2-dimethylsuccinate) | 1.07 | 1.60 | 1.04 | 0.325 | 0.237 |
| TSB46: Hederagenin *bis*-(3,3-dimethylglutarate) | 1.03^a^ | 0.94^a^ | 1.28^b^ | 0.070 | 0.007 |
| TSB47: Hederagenin *bis*-adipate | 1.03 | 1.14 | 1.01 | 0.128 | 0.570 |
| TSB50: Hederagenin-*bis*-(diglycolate) | 1.03 | 0.96 | 0.87 | 0.089 | 0.289 |
| TSB51: Hederagenin *bis*-(diglycinate) | 1.03 | 0.85 | 0.89 | 0.072 | 0.105 |
| TSB52: Hederagenin *bis*-(3,3-dimethylsuccinate) | 1.03^b^ | 0.81^a^ | 0.84^a^ | 0.069 | 0.041 |
| TSB58: Hederagenin *bis*-L-tartrate monomethyl ester | 1.03^b^ | 0.96^ab^ | 0.83^a^ | 0.063 | 0.047 |
|  |  |  |  |  |  |
| **Cholesterol and Cholic acid derivatives** |  |  |  |  |  |
| TSB39: Cholesteryl succinate | 1.95 | 2.05 | 1.95 | 0.211 | 0.865 |
| TSB40: Cholic succinate | 1.07 | 1.41 | 1.48 | 0.140 | 0.056 |
| TSB41: Cholic tri-succinate | 1.07 | 1.34 | 1.31 | 0.139 | 0.185 |
| TSB42: Lithocholic succinate | 1.95^a^ | 2.27^ab^ | 2.46^b^ | 0.159 | 0.047 |
| TSB43: Chenodesoxycholic bis-succinate | 1.07 | 1.53 | 1.85 | 0.286 | 0.087 |

^a-b^Means with different superscript differ (n=4)
