# Supplementary material for: Antiprotozoal Effect of Saponins in the Rumen Can Be Enhanced by Chemical Modifications in Their Structure
Source: Front Microbiol. 2017 Mar 16;8:399. doi: 10.3389/fmicb.2017.00399 (PMC5361656; doi:10.3389/fmicb.2017.00399)
Supplement: Supplementary file 3 [file Table_3.DOCX]

Table S3. Effect of hederagenin and bile acid derivatives, added at 0.5 or 1 g/L, on Isovalerate (%) after 24 h of incubation (batch culture)

|  | Dose g/L | | |  |  |
| --- | --- | --- | --- | --- | --- |
|  | 0 | 0.5 | 1 | SED | P |
|  | Isovalerate % of total VFA | | |  |  |
| Hederoside B | 0.87 | - | 0.84 | 0.106 | 0.749 |
|  |  |  |  |  |  |
| **Hederagenin derivatives** |  |  |  |  |  |
| TSB24: Hederagenin *bis*-succinate | 1.05^b^ | 0.94^ab^ | 0.82^a^ | 0.055 | 0.018 |
| TSB33: Hederagenin *bis*-(methylethylenglycolacetate) | 0.98 | 0.95 | 0.92 | 0.029 | 0.15 |
| TSB34: Hederagenin *bis*-(MeO-PEG4-carbonate) | 0.98 | 0.89 | 0.86 | 0.026 | 0.008 |
| TSB35: Hederagenin *bis*-glutarate | 0.98 | 0.90 | 0.97 | 0.055 | 0.348 |
| TSB36: Hederagenin *bis*-glycincarbamate | 0.98 | 0.92 | 0.89 | 0.040 | 0.131 |
| TSB37: Hederagenin *bis*-betainate dichloride | 0.98 | 0.87 | 0.87 | 0.059 | 0.166 |
| TSB38: Hederagenin *bis*-sulfate disodium salt | 1.12^b^ | 0.99^ab^ | 0.94^a^ | 0.052 | 0.034 |
| TSB44: Hederagenin *bis*-lactate | 0.88 | 0.94 | 0.825 | 0.086 | 0.426 |
| TSB45: Hederagenin *bis*-(2,2-dimethylsuccinate) | 0.88 | 0.77 | 0.80 | 0.069 | 0.337 |
| TSB46: Hederagenin *bis*-(3,3-dimethylglutarate) | 1.05^b^ | 0.74^a^ | 0.73^a^ | 0.059 | 0.003 |
| TSB47: Hederagenin *bis*-adipate | 1.05^b^ | 0.77^a^ | 0.76^a^ | 0.069 | 0.010 |
| TSB50: Hederagenin-*bis*-(diglycolate) | 1.05^b^ | 0.83^a^ | 0.75^a^ | 0.042 | 0.001 |
| TSB51: Hederagenin *bis*-(diglycinate) | 1.05^b^ | 0.84^a^ | 0.73^a^ | 0.053 | 0.003 |
| TSB52: Hederagenin *bis*-(3,3-dimethylsuccinate) | 1.05^b^ | 0.78^a^ | 0.74^a^ | 0.058 | 0.004 |
| TSB58: Hederagenin *bis*-L-tartrate monomethyl ester | 1.05^c^ | 0.90^b^ | 0.74^a^ | 0.040 | <0.001 |
|  |  |  |  |  |  |
| **Cholesterol and Cholic acid derivatives** |  |  |  |  |  |
| TSB39: Cholesteryl succinate | 1.15 | 1.18 | 1.19 | 0.062 | 0.847 |
| TSB40: Cholic succinate | 0.88 | 0.99 | 0.98 | 0.082 | 0.396 |
| TSB41: Cholic tri-succinate | 0.88 | 0.96 | 1.00 | 0.064 | 0.213 |
| TSB42: Lithocholic succinate | 1.15^b^ | 1.10^ab^ | 1.02^a^ | 0.035 | 0.028 |
| TSB43: Chenodesoxycholic bis-succinate | 0.88^a^ | 0.99^b^ | 1.15^b^ | 0.076 | 0.031 |

^a-b^Means with different superscript differ (n=4)
